# Effects of Saturated Soil Moisture on Fall Armyworm Pupal Development

**DOI:** 10.3390/insects16050521

**Published:** 2025-05-14

**Authors:** Tianqi Tian, Yingyan Zhai, Zhijie Chen, Yiwei Yang, Bo Hong

**Affiliations:** Shaanxi Key Laboratory of Qinling Ecological Security, Bio-Agriculture Institute of Shaanxi, Shaanxi Academy of Sciences, Xi’an 710043, China; tiantianqi@xab.ac.cn (T.T.); zhaiyy@xab.ac.cn (Y.Z.); zhijiechen68@xab.ac.cn (Z.C.); yangyw@xab.ac.cn (Y.Y.)

**Keywords:** *Spodoptera frugiperda*, developmental duration, emergence, IPM

## Abstract

*Spodoptera frugiperda*, known as the fall armyworm (FAW), a major invasive pest in corn, is rapidly spreading all over the world. Similarly to other Lepidoptera insects, FAW pupae usually develop in soil. Therefore, the soil moisture level is expected to be an important factor impacting their growth. In order to study the development and emergence of FAW pupae in a 100% soil moisture environment, three factors were selected for experiments in this study: the duration of saturated (100%) moisture treatment (0 h, 24 h, 48 h, and 72 h), the initial soil moisture before the larvae entered the soil (0 and 50%), and pupal age (1 day, 4 days, and 7 days). We discovered that (1) the emergence percentage of FAW pupae decreased with an increase in the saturated soil moisture treatment time, and the emergence percentage dropped to 0 after 72 h of continuous treatment; (2) the younger the age of FAW pupae, the more susceptible they were to being affected by saturated soil moisture treatment, and the emergence percentage of 7-day-old pupae was higher than that of 1-day-old pupae; and (3) FAW larvae that pupated in dry soil (0% moisture) had pupae with higher survival rates under subsequent 100% soil moisture stress, whereas those pupating in moderately moist soil (50% moisture) had lower survival rates under the same condition. Our study showed that the initial moisture level of the soil and the length of time the soil is saturated have a significant impact on FAW pupal development. The three factors of excessive stress time, wet initial soil moisture (50%), and lower pupal age ultimately lead to a decrease in the emergence percentage and survival rate of FAW pupae.

## Simple Summary

The fall armyworm (FAW) is an important agricultural pest that is especially harmful to corn. Among the control strategies that have been applied, irrigation has been mentioned multiple times and is speculated to be an effective method of reducing pupal populations in the soil. However, it is not yet clear whether this method is effective in controlling the fall armyworm. In this study, we designed a crossover experiment with multiple factors (including pupal age, duration of saturated soil moisture, and initial soil moisture before pupation) to investigate the survival ability of fall armyworm pupae in a saturated soil moisture environment under different conditions. We found that increasing the duration of saturated soil moisture is detrimental to the survival of FAW pupae, but a higher pupal age and larvae pupating in dry soil are beneficial to the survival of FAW pupae. The results of this study provide a valuable reference for formulating pest control strategies for the fall armyworm.

## 1. Introduction

*Spodoptera frugiperda* (Smith), which is also known as the fall armyworm (FAW), belonging to Lepidoptera, Noctuidae, is a type of phytophagous insect originating in the tropical and subtropical Americas [1]. The FAW is considered an important agricultural pest [2], known to damage over 353 plant species, with corn being its preferred host [3]. According to statistics, the economic losses caused by the FAW in corn production are estimated to be between 15% and 73% globally [4,5]. The fall armyworm was first reported in West Africa in January 2016, and since then, this species has begun to spread at an alarming rate [6,7]. Within only two years, traces of fall armyworm activity had been detected in 28 sub-Saharan African countries [8,9]. Since 2018, the FAW has invaded the Middle East [10,11,12], South Asia [13,14], Southeast Asia [15,16,17,18], East Asia [19,20,21,22], Oceania [23], and Europe [24,25]. At present, the threat posed by the FAW to global corn production is still increasing [2]. Considering their rapidly intensifying economic impacts [26], studies on the various behaviors of FAWs throughout their life cycle can contribute to practical applications in FAW management, and these studies are attracting increasing attention [27].

The life cycle of the fall armyworm has been carefully studied in different hosts and regions [28,29]. After the larvae of the FAW grow to the last instar, they will leave the host plant, burrow into the soil, build a pupal chamber, complete pupation, and continue to develop into adults in the soil [30]. Therefore, the development process of FAW pupae in soil is one of the most important parts in its whole life cycle. It is worth noting that abiotic factors in soil have been widely recognized as having a significant impact on insects that spend part of their life cycle in soil [31,32,33]. For example, soil moisture can affect pupa formation from larvae and adult emergence processes in various Lepidoptera species [34,35,36]. Similarly to other Lepidoptera species, the fall armyworm is affected by soil moisture during the larval and pupal stages. A study has demonstrated that higher soil moisture can significantly inhibit the pupation behavior of FAWs [37], and other studies have shown that a soil moisture level not exceeding 80% has no effect on the survival and emergence of FAW pupae [38]. In addition, a study by Xu’s team also found that flooding had an adverse effect on the emergence of FAW pupae and reproduction in adults [39]. Based on these results, an IPM strategy was proposed that includes methods such as irrigation to exterminate pupae in the soil and control FAW populations in corn cultivation. However, the ability of FAW pupae to survive flooding may be affected by a variety of factors, including pupal age and different pupal environments. Current research predominantly focuses on isolated factor analyses, which may not provide a comprehensive assessment, given the inherent diversity and complexity of environmental stressors. Critically, the combined effects of these factors on the survival of FAW pupae have not been discussed or studied academically. Therefore, the response of FAW pupae to flooding requires further study.

Against this background, we conducted a series of cross-factor experiments. These factors included pupal age, the duration of saturated moisture treatment, and the initial soil moisture before larvae pupation. We studied their effects on the development and survival of FAW pupae in environments with extreme soil moisture, including (1) the emergence of FAW pupae, (2) whether FAW pupae of different pupal ages showed different resistance to 100% soil moisture, and (3) whether the soil moisture level during the pupation of FAW larvae affected the emergence percentage of pupae with 100% soil moisture. Understanding the impact of 100% soil moisture on Lepidoptera pupae represents a continuation of our previous research work [40] and will provide valuable insights into the response of Lepidoptera pupae to soil moisture and contribute to the practical application of IPM strategies in FAW control.

## 2. Materials and Methods

### 2.1. Insects

The tested FAW larvae were obtained from the corn plants cultivated in the experimental field (109.84° E, 34.76° N) of Bio-Agriculture Institute of Shaanxi, Shaanxi Academy of Sciences, Dali County, Shaanxi Province, China. To avoid the cannibalism of FAW mature larvae [41], we reared larvae individually in compartments of plastic containers (16 × 22 × 8 cm) and fed them 300 g of fresh corn leaves per day. Unless otherwise stated, all experiments were performed under conditions of L:D = 14 h:10 h, 27 ± 2 °C, and 75 ± 10% relative humidity. Before the experiment, the last-instar larvae, within 12 h of molting, were transferred to a square glass container (1.5 × 10 × 10 cm) and provided with corn leaves every 24 h.

### 2.2. Soil Moisture Treatment

Experimental soil (loam, 47.6% sand, 28.3% silt, and 24.1% clay; pH = 7.25; organic matter content = 6.1%) was collected from a field of the Bio-Agriculture Institute of Shaanxi, Dali County, Shaanxi Province. Soil samples were collected using the core method with a hammer sampler at a depth of 5–10 cm to preserve the bulk density (1.35 g/cm^3^), soil particle density (2.63 g/cm^3^), and porosity (50.2%). We first treated the soil in an oven at 50 °C for 7 days until it was completely dry. Then, we crushed the dried large clods and, with a 2 mm sieve, sieved and rerolled them. Finally, we added different amounts of distilled water to the dry soil to create different soil moisture conditions. Soil moisture = (weight of added distilled water)/(saturated soil weight-dry soil weight) × 100%. The saturated soil weight here refers to the situation where all the pores in the soil are completely filled with water, that is, the condition where there is no air in the soil. The saturated soil weight was measured using the following method [42]: (1) Weigh a cutting ring (short cylindrical steel tube with one sharp edge, volume 100 cm^3^) and use this cutting ring to collect soil. Then, install a bottom with small holes on the cutting ring and place a layer of filter paper on the inside of the bottom to prevent soil particles from leaking out through the small holes. (2) Place the cutting ring with soil in the water, keeping the water level with the top of the cutting ring but not flooding over the top edge. Make sure that the water is slowly absorbed into the soil from the bottom through the capillary phenomenon. (3) Soak the cutting ring for 24 h and then take it out. Wipe the water from the outer surface of the cutting ring and then weigh the cutting ring set. After weighing and recording, put the cutting ring set back into the water to continue soaking and absorbing water. (4) Repeat step 3 above until the weight of the cutting ring set is constant. At this time, the saturated moisture soil weight = total weight—cutting ring weight.

According to previous research [40], we chose two different soils, namely dry (i.e., 0% soil moisture) and wet (i.e., 50% soil moisture), for larvae to pupate in. The soil moisture at this time was defined as the “initial soil moisture”. In order to observe the process of the pupal stage, we designed a square glass container (1.5 × 10 × 10 cm) with freely switchable drainage holes at the bottom and transferred the last-instar larvae within 12 h of molt into the container. The container was filled with soil with the corresponding moisture level (0% and 50%), and every 24 h, we fed the larvae with 30 g of corn leaves. Briefly, we transferred the larvae to a 1.5 cm wide glass container of soil to pupate and placed the soil between two pieces of glass (10 cm high) to create more convenient observation conditions.

### 2.3. Effect of Saturated Soil Moisture on Pupal Survival and Emergence

Last-instar larvae were placed in glass containers with soil with a set initial humidity, with one larva in each container, and left to reach pupation naturally. Considering that different initial soil moisture levels will affect the pupation rate of larvae [31], in order to avoid different pupation rates influencing the results of this experiment, we only selected samples that successfully pupated for consideration in the experiment. According to the development time of FAW pupae at 27 °C [43], three different ages (1 day, 4 days, and 7 days) were selected for the experiments. We added distilled water to the soil along the edge of the container at different times so that the soil moisture reached 100%, and then, we maintained it for 0 h, 24 h, 48 h, and 72 h, respectively. In order to maintain the corresponding soil state, we monitored water evaporation by weighing every 24 h and adding appropriate amounts of distilled water to the container according to the monitoring results. After each treatment, we opened the drainage holes at the bottom of the container and placed it on completely dry soil to allow the water to seep out quickly. At the same time, we kept the environment ventilated and reduced the soil moisture from saturation within 12 h and then continued to reduce it to the optimal level for FAW pupal development (50% soil moisture) [37]. After that, we closed the container drainage holes; we still kept the soil moisture level steady by weighing and adding water every 24 h until all samples in the experiment had either eclosed or died.

Through observation, we monitored the pupal development and emergence process in the laboratory every day and recorded the pupal development time. In addition, we were able to calculate the percentage of emergence (number of adults that emerged/number of pupations) by recording the number of adults that emerged. The experiment was carried out under the conditions of L/D time 14 h/10 h, temperature 27 ± 2 °C, and relative air humidity 75 ± 10%. There were 24 treatment combinations in the experiment, and each treatment was performed 5 times with 30 insects each time.

### 2.4. Data Analysis

Descriptive statistics are given as the mean and standard error of the mean. Prior to parametric analyses, data distributions were rigorously evaluated through Shapiro–Wilk tests for normality and Levene’s test for the homogeneity of variances. When parametric assumptions were violated, dependent variables were subjected to base-10 logarithmic transformation (applied to both the pupal development time and adult emergence percentage) to achieve approximate normality and variance stabilization [44], with the subsequent reconfirmation of assumption compliance using the same diagnostic tests. Reported means ± SEM in figures and tables were back-transformed to the original scale for clarity.

We used three-way and two-way ANOVAs to examine the main and interactive effects of the initial soil moisture, treatment duration, and pupal age on the pupal development time and percentage of adult emergence in the FAW, using the general linear model procedure of SPSS 23.0 (SPSS, Chicago, IL, USA). When statistically significant second-order interactions (two-way) or third-order interactions (three-way) were identified, we conducted stratified simple effects analyses through one-way ANOVAs followed by Bonferroni-adjusted pairwise comparisons to isolate specific group differences.

## 3. Results

### 3.1. Effect of Saturated Moisture Treatment Duration on the Emergence Percentage in FAW Pupae

**Figure 1:**
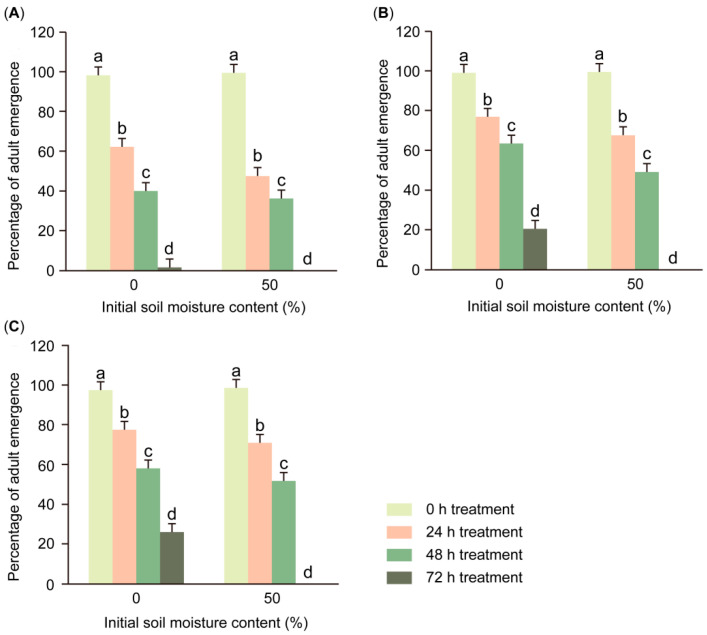
Percentages of FAW adult emergence in 1-day-old pupae (**A**), 4-day-old pupae (**B**), and 7-day-old pupae (**C**) after various durations of saturated moisture treatment and with various initial soil moisture levels (mean ± SE). Statistical comparisons were conducted separately within each initial soil moisture level. Different letters above the bars indicate significant differences among treatments (mean separation using Bonferroni correction, *p* < 0.05); bars sharing the same letter are not significantly different.

The duration of 100% soil moisture (*F*_3,96_ = 1371.796, *p* < 0.001) had a significant effect on the emergence percentage in pupae at different pupal ages (Table 1), and with the increase in treatment time, the emergence percentage in pupae showed a decreasing trend (Figure 1). Compared with the untreated group (treated 0 h), the emergence percentage in FAW pupae at all pupal ages was significantly reduced after being treated with 100% soil moisture for at least 24 h (1-day-old pupae and 0 initial soil moisture, *F*_3,32_ = 251.030, *p* < 0.001; 1-day-old pupae and 50% initial soil moisture, *F*_3,32_ = 258.474, *p* < 0.001; 4-day-old pupae and 0 initial soil moisture, *F*_3,32_ = 300.349, *p* < 0.001; 4-day-old pupae and 50% initial soil moisture, *F*_3,32_ = 472.328, *p* < 0.001; 7-day-old pupae and 0 initial soil moisture, *F*_3,32_ = 108.161, *p* < 0.001; 7-day-old pupae and 50% initial soil moisture, *F*_3,32_ = 202.888, *p* < 0.001). Among all the experimental groups, the 1-day-old pupae that entered the soil at 50% initial soil moisture showed the highest decline in emergence percentage after 24 h of treatment at 100% soil moisture, from 99.3 ± 1.5% (treated for 0 h) to 47.3 ± 4.3%. After continuous saturated moisture treatment for 72 h, the emergence percentage in 1-day-old, 4-day-old, and 7-day-old pupae that entered the soil at 0 initial soil moisture decreased to 1.3 ± 3.0%, 20.7 ± 2.8%, and 26.0 ± 10.9%, respectively. After the same treatment, the emergence percentages in 1-day-old, 4-day-old, and 7-day-old pupae that entered the soil at 50% initial soil moisture all decreased to 0.

### 3.2. The Effect of Pupal Age on the Emergence of FAW Pupae

**Figure 2:**
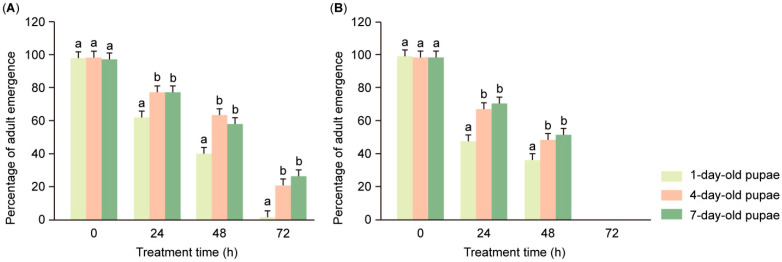
Percentages of FAW adult emergence in the dry soil (**A**) and with 50% initial soil moisture (**B**) at various days of age in pupae and various saturated moisture treatments (mean ± SE). Statistical comparisons were conducted separately within each treatment time. Different letters above the bars indicate significant differences among treatments (mean separation using Bonferroni correction, *p* < 0.05); bars sharing the same letter are not significantly different.

The results show that pupal age (*F*_2,96_ = 58.026, *p* < 0.001) had a significant effect on the emergence percentage of pupae in a 100% soil moisture environment (Table 1) and that the emergence percentage increased with pupal age (Figure 2). For the pupae that entered into the soil at an initial soil moisture of 0, the emergence percentage in 1-day-old pupae after treatment for 24 h, 48 h, and 72 h was significantly lower than that in 7-day-old pupae treated at the same time (treated for 24 h, *F*_2,48_ = 9.510, *p* < 0.001; treated for 48 h, *F*_2,48_ = 18.139, *p* < 0.001; treated for 72 h, *F*_2,48_ = 20.440, *p* < 0.001; Figure 2A). For the pupae that entered the soil at 50% initial soil moisture, the emergence percentage in 1-day-old pupae treated for 24 h and 48 h, respectively, was also significantly lower than that in 7-day-old pupae treated for the same time (24 h, *F*_2,48_ = 37.885, *p* < 0.001; 48 h, *F*_2,48_ = 15.965, *p* < 0.001; Figure 2B).

### 3.3. The Effect of Initial Soil Moisture on the Emergence Percentage in FAW Pupae

**Figure 3:**
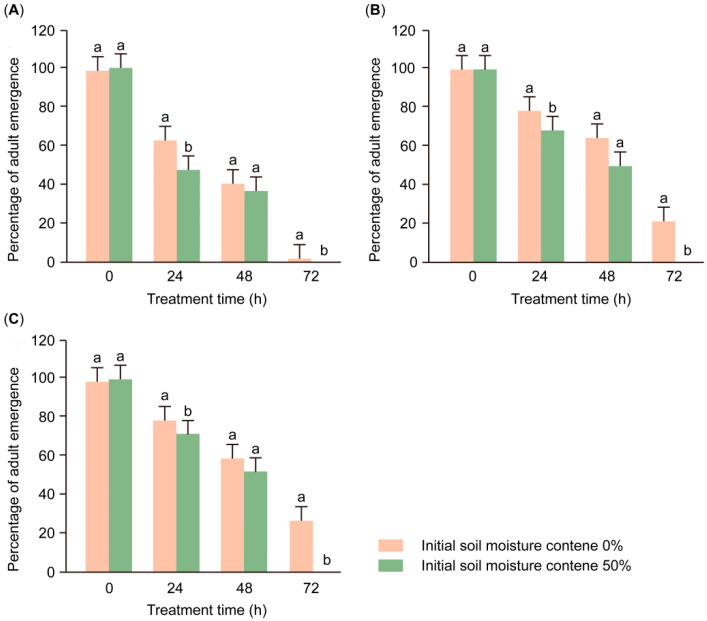
Percentages of FAW adult emergence for groups of 1-day-old pupae (**A**), 4-day-old pupae (**B**), and 7-day-old pupae (**C**) for various initial soil moistures and saturated moisture treatment durations (mean ± SE). Statistical comparisons were conducted separately within each treatment time. Different letters above the bars indicate significant differences among treatments (mean separation using Bonferroni correction, *p* < 0.05); bars sharing the same letter are not significantly different.

The experimental results show that the initial soil moisture (*F*_1,96_ = 69.669, *p* < 0.001) had a significant effect on the emergence percentage in FAW pupae (Table 1). After 24 h of saturated moisture treatment, the emergence percentage in FAW 1-day-old pupae that entered the soil at the initial soil moisture (62.0 ± 6.0%) was significantly higher than that in FAW 1-day-old pupae that entered the soil at 50% initial soil moisture (47.3 ± 4.3%) (*F*_1,32_ = 16.477, *p* < 0.001; Figure 3A). After 72 h of saturated moisture treatment, the emergence rates of 4-day-old and 7-day-old FAW pupae that entered the soil at an initial 50% soil moisture decreased to 0. In contrast, these data are significantly lower than the emergence percentage for 4-day-old (20.7 ± 2.8%; *F*_1,32_ = 59.138, *p* < 0.001; Figure 3B) and 7-day-old FAW pupae (26.0 ± 10.9%; *F*_1,32_ = 39.635, *p* < 0.001; Figure 3C) that entered the soil at an initial soil moisture of 0.

### 3.4. The Effect of Saturated Moisture Treatment Duration, Pupal Age, and Initial Soil Moisture on Pupal Development Duration in FAW Pupae

There were no main or interactive effects from initial soil moisture, saturated moisture treatment duration, and pupal age on pupal development duration in FAWs (Table 2). In all experimental groups, the longest pupal development duration was 10.7 ± 0.1 d (4-day-old pupae that entered the soil at an initial soil moisture of 0, with saturated moisture treatment for 24 h); the shortest was 10.4 ± 0.2 d (4-day-old pupae that entered soil at initial soil moisture of 50%, with saturated moisture treatment for 48 h).

## 4. Discussion

Similarly to the pupae of other Lepidoptera insects [45,46,47], the last-instar larvae of FAWs dig holes in the soil to select a location and build a pupal cell as a suitable place for pupation. Previous studies have shown that the pupal chamber can have a certain protective effect on pupae; for example, it can improve the survival rate of hibernating pupae in soil [48,49]. Given the physical properties of the pupal cell, soil particles and silk can protect FAW pupae from external environmental changes. The pupal chamber is likely to provide an isolation layer, alleviating temperature and water changes through the action of soil particles and silk and establishing a water-stable microenvironment to protect FAW pupae from water stress. It has been shown that the percentage of FAW adult emergence is not affected by soil moisture when the soil is not saturated [36,38]. This is partly because the pupal chamber provides insulation and helps buffer changes in moisture [40]. However, we found that 100% soil moisture is a threat factor in the survival of FAW pupae. As the duration of saturated moisture treatment increased, the emergence percentage of FAW pupae decreased significantly (Figure 1). Some existing studies have also supported this conclusion [38,50]. Additionally, Xu found that FAW pupae failed to emerge after 4 days of waterlogging [39], which is similar to our result (72 h). Through observation, we found that when the water content in the soil reaches saturation (100% soil moisture), the water gradually infiltrates and fills the pupal cell so that the pupa is completely submerged. Therefore, the protection afforded by the pupal compartment may be limited when faced with more extreme, prolonged water stress. High soil moisture can have an impact on the properties of the soil environment, including soil respiration and CO_2_ and O_2_ concentration levels [51], and the continuous hypoxia caused by a decrease in oxygen solubility may be the main reason behind the decrease in the pupal emergence percentage [52].

According to our experimental results, the emergence percentage increased with pupal age (Figure 2). Under the same treatment conditions, the emergence percentage in 1-day-old pupae was always the lowest, while that in 7-day-old pupae was the highest. This indicates that newly formed pupae were more susceptible to high soil moisture levels, which adversely affected emergence, compared to older pupae. Similarly to FAW pupae, when the pupae of some Lepidoptera and Diptera insects develop under high soil moisture, an increase in pupal age will also significantly increase the emergence percentage, such as in the cotton bollworm (*Helicoverpa armigera* (Hübner)) and peach fruit fly (*Bactrocera zonata* (Saunders)) [53,54]. These data may serve as a support for our results, but the mechanism has not been clearly reported and studied.

The initial soil moisture level in which FAW larvae pupate also affects the emergence percentage. The results showed that after flooding, the emergence percentage of FAWs that pupated in dry soil (0 soil moisture) was always higher than that of FAWs that pupated in wet soil (50% soil moisture) (Figure 3). In a previous study [40], we found that soil moisture during larval pupation significantly affects the depth at which larvae dig holes, build pupal chambers, and bury pupae. When the soil is too dry, the construction of the pupal chamber takes more time for the larvae, and the depth of the pupal chamber (0.5 cm on average) is much shallower than that achieved under the appropriate soil moisture level (2.5 cm on average) [40]. However, while dry soil adversely affects larval burrowing behavior, the shallower depth of pupal burial reduces the likelihood of complete submersion in water under conditions of 100% soil moisture. This results in improved ventilation for the pupae and indirectly improves the resistance of pupae to the stress of high soil moisture.

Under all tested conditions, the emergence rate significantly declined with increasing saturated moisture treatment duration, but the extent of this decline was modulated by both the initial soil moisture and pupal age. Specifically, under 50% initial soil moisture combined with ≥72 h of saturated moisture treatment, the emergence rate dropped to 0 across all pupal ages, indicating that high initial soil moisture and prolonged saturation exert a synergistic negative effect on pupal survival. In contrast, with the same treatment duration but 0% initial soil moisture, a proportion of pupae still successfully emerged (e.g., 26.0 ± 10.9% in 7-day-old pupae), suggesting greater tolerance under drier initial conditions. Furthermore, within the 24–48 h saturated moisture exposure range and 0 initial soil moisture, older pupae (≥4 days) exhibited notably higher emergence rates, whereas this age-related advantage was diminished when initial soil moisture was 50%, resulting in overall low survival. These observations demonstrate that the survival of FAW pupae in waterlogged environments is not governed by a single factor but arises from complex interactions among treatment duration, initial soil moisture, and pupal developmental stage.

Additionally, these interactions exhibit nonlinear and threshold-like behavior. When multiple unfavorable conditions coincide, such as high initial soil moisture and extended saturation, they may override otherwise favorable factors such as pupal age. Conversely, under less extreme conditions, factors like older pupal age may still confer increased resilience. Therefore, assessing pupal emergence or mortality based on any single factor in isolation may lead to inaccurate predictions, especially in field applications where multiple stressors coexist.

In our experiment, 100% soil moisture did not significantly increase the developmental duration of FAW pupae, and there was no significant difference in pupal developmental duration under each treatment (Table 2). Excess soil moisture has been confirmed as having a “hysteresis effect” on pupal stage development (i.e., developmental progress slows down as soil moisture increases), possibly because hypoxia prolongs pupal development in FAWs [48]; however, under our experimental conditions, sudden short-term (≤72 h) flooding does not seem to be sufficient to cause a significant increase in developmental duration. The detailed mechanism may require further study.

## 5. Conclusions

This study demonstrated that the survival of FAW pupae under saturated (100%) soil moisture conditions is jointly regulated by treatment duration, initial soil moisture, and pupal age. Emergence rates declined with prolonged saturation, especially when pupae originated from moist soils (50% moisture) or were at younger developmental stages. Pupae from dry soils (0% moisture) showed greater tolerance to moisture stress. Although older pupae exhibited higher survival under short-term stress (24–48 h), this advantage diminished when initial soil moisture was high.

These findings have important implications for pest management. Maintaining saturated soil conditions for more than 72 h through irrigation could effectively suppress FAW pupal populations, similar to the successful control of *Chilo suppressalis* using spring irrigation [55,56]. Irrigation strategies could not only reduce pest numbers but also save water and improve crop yields, aligning with the recommendations of agricultural scientists [54,57,58,59]. However, the effectiveness of such strategies may vary under arid or semi-arid conditions where pupae develop in dry soils, potentially reducing the mortality effect [60]. Furthermore, soil physical properties (such as texture, bulk density, and porosity) critically influence soil water retention [61,62,63,64,65,66] and thus the persistence of high moisture conditions, necessitating field validation under diverse environmental contexts.

## Figures and Tables

**Table 1:** Factorial ANOVA results for effects of initial soil moisture, saturated moisture treatment duration, and pupal age on emergence percentage in adult *S. frugiperda*.

Source	ANOVA	df	Mean Square	*F*-Value	*p*-Value
Initial soil moisture	One-way	1	0.217	69.669	<0.001
Saturated moisture treatment duration	One-way	3	4.267	1371.796	<0.001
Pupal age	One-way	2	0.181	58.026	<0.001
Initial soil moisture × Saturated moisture treatment duration	Two-way	3	0.037	11.907	<0.001
Initial soil moisture × Pupal age	Two-way	2	0.012	3.812	0.025
Saturated moisture treatment duration × Pupal age	Two-way	6	0.026	8.260	<0.001
Initial soil moisture × Saturated moisture treatment duration × Pupal age	Three-way	6	0.014	4.514	<0.001
Error		96	0.003		

**Table 2:** Factorial ANOVA results for effects of initial soil moisture, saturated moisture treatment duration, and pupal age on pupal development duration in *S. frugiperda*.

Source	ANOVA	df	Mean Square	*F*-Value	*p*-Value
Initial soil moisture	One-way	1	0.054	1.653	0.202
Saturated moisture treatment duration	One-way	3	0.058	1.774	0.159
Pupal age	One-way	2	0.003	0.096	0.908
Initial soil moisture × Saturated moisture treatment duration	Two-way	2	0.003	0.102	0.903
Initial soil moisture × Pupal age	Two-way	2	0.017	0.532	0.590
Saturated moisture treatment duration × Pupal age	Two-way	6	0.025	0.767	0.598
Initial soil moisture × Saturated moisture treatment duration × Pupal age	Three-way	4	0.012	0.377	0.824
Error		80	0.033		

## Data Availability

Data supporting the information shown in the results are openly available in a public repository at https://zenodo.org/records/14557197 (accessed on 20 December 2024).
